# Measurement of the interplay effect in lung IMRT treatment using EDR2 films

**DOI:** 10.1120/jacmp.v7i4.2222

**Published:** 2006-11-28

**Authors:** Ross I. Berbeco, Cynthia J. Pope, Steve B. Jiang

**Affiliations:** ^1^ Department of Radiation Oncology Massachusetts General Hospital and Harvard Medical School Boston Massachusetts U.S.A.; ^2^ Department of Radiation Oncology Dana‐Farber/Brigham and Women's Cancer Center and Harvard Medical School Boston MA U.S.A.

**Keywords:** radiotherapy, IMRT, organ motion, lung treatment, interplay effect

## Abstract

Intrafraction organ motion during the dynamic delivery of intensity‐modulated radiation therapy (IMRT) treatment of lung tumors may cause unexpected hot/cold spots within the target volume, due to the interplay effect between tumor motion and multileaf collimator (MLC) leaf motion. In the past, this has been investigated through theoretical analysis, computer simulation, and experimental measurement using an ionization chamber dosimeter. In the work presented here, the interplay effect was studied experimentally in 2D, using Kodak EDR2 films. A five‐field lung IMRT plan was delivered to a solid water phantom with embedded film. The phantom was placed on a motor‐driven platform with a sinusoidal motion to simulate the respiration‐induced tumor motion. The delivery of each field began at one of eight equally spaced initial breathing phases. The dose distribution for each treatment fraction was estimated by combining the dose distributions for all fields with randomly sampled initial breathing phases. The dose variation caused by the interplay effect was estimated by looking at the dose values from 1000 trials of 30 fractions. It was found that, on a day‐to‐day basis, the standard deviation of the dose to a given pixel in the high‐dose region could be as high as 2% to 4% due to the motion interplay effect. After 30 fractions, the standard deviation in the dose to each pixel is reduced to 0.4% to 0.7%. However, compared to the static delivery, the dose distribution from a 30‐fraction case in the presence of motion shows some underdosing in the region of interest. We found that the maximum dose in the target remains within 1% of the maximum dose in the static case, but the minimum dose in the target is most likely to be about 6% lower than the static case. Our results indicate that there can be some underdosing of the tumor due to the interplay effect in lung IMRT delivery over the entire course of a 30‐fraction treatment.

PACS number: 87.53.Mr

## I. INTRODUCTION

The goal of radiotherapy treatment is the placement of a high dose within the target area(s) with minimal dose accumulated in healthy tissues and organs. A proven method for improving the conformality of a dose distribution is intensity modulated radiation therapy (IMRT).^(^ ^1^ ^–^ ^4^ ^)^ Clinicians are hesitant to treat non‐small‐cell lung cancer patients with IMRT, even though clinical studies have demonstrated that an escalation in radiation dose results in significant improvement in the outcome of these patients.^(^ ^5^ ^,^ ^6^ ^)^ The reluctance is due to organ motion effects, including dose blurring effect and interplay effect.^(7)^

The blurring effect is a convolution of the dose distribution delivered to a static target with a motion kernel and is, consequently, an issue for any delivery technique. Dose blurring is most important at the edges of the treatment volume, where the dose gradient is high. Therefore, a sufficient safety margin should be used.

The interplay effect is a combination of tumor motion and beam motion (as it is shaped by a dynamic multileaf collimator (MLC)) and has been studied both theoretically and experimentally.^(^ ^7^ ^–^ ^16^ ^)^ This effect is only an issue for dynamic delivery techniques (including step‐and‐shoot IMRT) and may cause hot/cold spots within the target as well as outside it. Previous experimental work used point measurements and/or a single beam only. This work expands on those earlier studies by using Kodak EDR2 film to estimate the delivered dose distribution in an axial plane in the presence of organ motion for a multifield, multifraction IMRT treatment.

## II. METHODS

**Figure 1:**
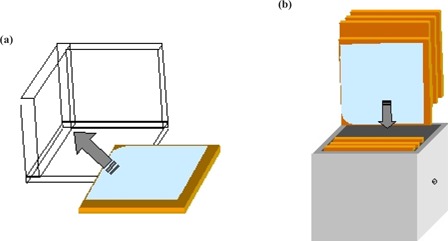
The film is aligned using a 3D square and then (b) placed in the NOMOS IMRT QA phantom.

To simulate patient breathing, we used a 24‐V DC motor‐driven platform. The platform moves sinusoidally, 2 cm peak‐to‐peak on rails (one translational degree of freedom) with a frequency that is determined by the current supplied to the motor. Although lung tumors tend to move in elliptical paths,^(17)^ the interplay effect is mostly due to motion perpendicular to the leaf motion (i.e., the cranial–caudal direction), so one‐dimensional motion in the S‐I direction is acceptable for our purposes. The construction material of the motion platform is mostly radio‐translucent plastic, so as not to affect the dose distribution. The period of oscillation of the platform was set to approximately 4 s to model typical patient breathing.^(^ ^17^ ^–^ ^19^ ^)^ For the irradiation, we placed a NOMOS IMRT QA phantom on the platform. Kodak EDR2 film was placed within the phantom in an axial orientation using a 3D square to ensure reproducible alignment (see Fig. 1). We irradiated the phantom at 500 MU/min using a five‐field IMRT plan with 20‐segment step‐and‐shoot delivery optimized with the Helios inverse‐planning system (Varian Medical Systems, Inc., Palo Alto, CA). The LINAC used for the experiment was a Varian 2100 C/D with a 120‐leaf MLC (Varian Medical Systems, Inc., Palo Alto, CA). The motion of the platform was set in the S‐I direction and the MLC leaves moving across the patient such that the two motions were always perpendicular. This was done to ensure consistency with a previously published study.^(7)^ As a control, each of the five fields was measured individually, as well as a single film containing all five fields, with the platform in a static state.

For the motion studies, the phantom was set up on the treatment table using the in‐room lasers. The platform motor was then turned on and the irradiation was performed beginning at one of eight equally spaced breathing phases for each field. The different starting phases were used to simulate a clinical situation in which the therapist turns the beam on at random starting phases. The phase was determined visually using a closed‐circuit video monitoring system. This was repeated for all five fields and for all eight phases to obtain a total of 40 films. The films were then scanned to digital format for analysis using a VXR‐16 16‐bit film scanner (Vidar Systems Corp. Herndon, VA) and the WP700 software (Scanditronix Wellhofer Bartell, TN).

Due to a feature of the scanner, the films had to been fed with one corner first (i.e., not with a flat edge leading). The angle of film during scanning varied; therefore, the digital images showed the field edges at different angles. For a proper comparison, the field images had to be rotated to the same orientation. A program was written in Interactive Data Language (IDL) (Research Systems Inc, Boulder, CO) to detect the field edges and reorient the 40 images. From our experience with the reorientation procedure, we estimate the error to be less than one pixel (1 mm) in any direction. Since this is a study of dose distributions and not single‐point measurements, the error associated with the field reorientation is negligible. In addition, the digital reorientation of the field helped correct for any manual misalignment in the initial placement of the film in the solid water.

## III. RESULTS AND DISCUSSION

**Figure 2:**
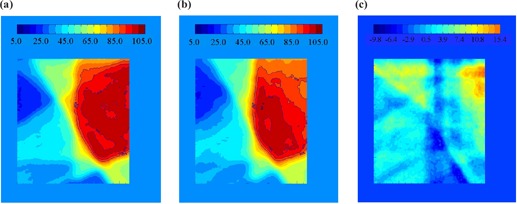
The combination of the five fields delivered to the static platform. For irradiations with the platform in motion, the initial phase was randomly selected. Image (b) is an example of one possible distribution when motion is present. Image (c) is the difference between the example motion distribution and the static distribution. The values of (c) range from −9.8% to +15.4% of the total prescribed dose.

**Figure 3:**
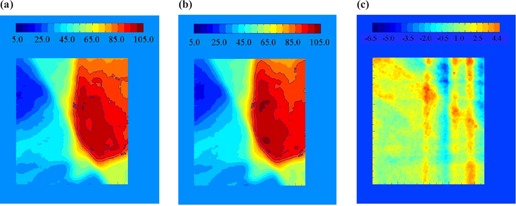
(a) and (b) show ${\text{TD}}_{1}^{\left\{\mu \right\}}$ for two different samples of initial motion phases, {μ}, of the platform. Subtracted from each other, (c) shows an example of the possible changes of the daily distribution delivered when motion is present. The values in this example range from −6.5% to +4.4% of the total prescribed dose.

**Figure 4:**
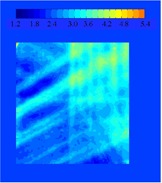
A plot of the value of the standard deviation (1σ) of ${\text{TD}}_{1}^{\left\{\mu \right\}}$ at each point in the array after 1000 trials of randomly selected motion phases. The values of the standard deviation range from 1.2% to 5.4%. Within the high‐dose region (90% isodose curve), the variation is 2.0% to 3.8%.

**Figure 5:**
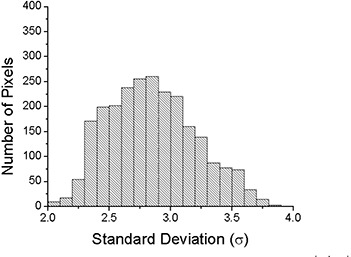
Histogram of the standard deviation of 1000 trials of the single‐ case. Only pixels with a value of at least 90% on the static dose plot were used to calculate this histogram.

Two quantities were studied:

(1) $${\text{TD}}_{1}^{\left\{\mu \right\}}=\sum_{j=1}^{5}D_{j\mu }$$

and

(2) $${\text{TD}}_{30}^{\left\{\mu \right\}}=\sum_{m=1}^{30}\sum_{j=1}^{5}D_{j\mu },$$

where ${\text{TD}}_{1}^{\left\{\mu \right\}}$ is the total dose distribution after a single fraction, and ${\text{TD}}_{30}^{\left\{\mu \right\}}$ is the total dose distribution after 30 fractions. Here, *j* represents the field (1 to 5), *m* is the fraction (1 to 30), and μ is a random integer between 1 and 8, representing the initial motion phase. {μ} is the set of μ's for a given calculation. The results can be subtracted from the static distribution, ${\text{TD}}_{S}$, to calculate the difference. An example of ${\text{TD}}_{S}-{\text{TD}}_{1}^{\left\{\mu \right\}}$ for a randomly selected μ is shown in Fig. 2. For the example shown, the values range from −9.8% to +15.4% of the total prescribed dose. Calculations of ${\text{TD}}_{1}^{\left\{\mu \right\}}$ for different starting phases can also be compared to each other. Figure 3 shows an example of the possible changes of the daily distribution delivered when motion is present. The values in this example range from −6.5% to +4.4% of the total prescribed dose. The latter calculation can be repeated many times with a random sampling of initial motion phases for the platform to find the standard deviation of a single fraction. The standard deviation of the dose distribution at each pixel for 1000 trials is shown in Fig. 4. The values of the standard deviation range from 1.2% to 5.4%. Within the high‐dose region (90% isodose curve), the variation is 2.0% to 3.9%. A histogram of the standard deviation within the 90% static isodose curve (the target area) is shown in Fig. 5. The peak of the distribution is around 2.8%.

**Figure 6:**
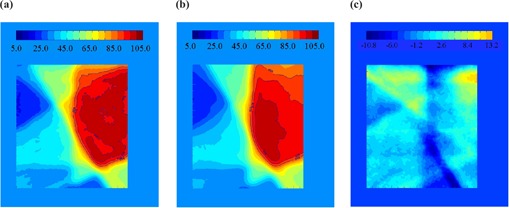
The sum of all five fields delivered to a static platform. (b) The average of 30 fractions of five fields delivered to a moving platform with randomly selected initial phases. (c) Some differences in the two distributions may still be seen after the averaging effects of 30 treatments.

**Figure 7:**
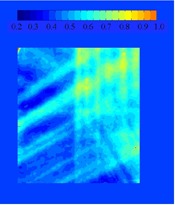
The standard deviation (1σ) from 1000 trials of the 30‐fraction case.

**Figure 8:**
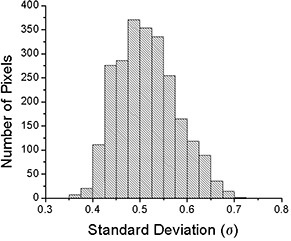
The histogram of the standard deviation in each pixel in the region of interest for the 30‐fraction case.

The same analysis was done for ${\text{TD}}_{30}^{\left\{\mu \right\}}$. Figure 6 shows the subtraction of an irradiation with a 30‐fraction sample of random initial motion phases, {μ}, from the static one. Some differences in the two distributions may still be seen after the averaging effects of 30 treatments. The standard deviation at each pixel for 1000 trials is shown in Fig. 7. Although there is a difference in the distribution of the 30‐fraction case and the static case (as was shown in Fig. 6), the standard deviation among the possible final dose distributions is reduced substantially after 30 fractions. The maximum standard deviation was reduced from 5.4% in the single fraction case to less than 1.0% after 30 fractions. A histogram of the standard deviation within the 90% static isodose curve is shown in Fig. 8.

The daily dose variation can be significant due to motion. The standard deviation of the differences from 1000 trials ranges from 1.2% to 5.4% over the entire film and from 2.0% to 3.8% within the region of interest (the 90% static isodose curve). However, after 30 fractions, the variation is reduced to less than 1%, and the range is between 0.36% and 0.71% within the region of interest. This is due to the canceling out of hot/cold spots when many fractions are combined. These results compare favorably with a previous experiment performed using an ion chamber for measurement.^(7)^ In that study, the standard deviation was 0.19% and 0.35% for two different patients for 30 fractions.

**Figure 9:**
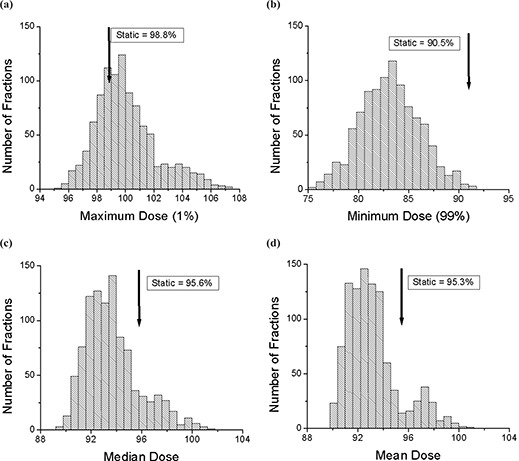
The histograms for 1000 trials of single‐fraction treatments with motion. All the values are calculated within the region of interest (static 90% isodose curve). (a) The maximum (99th percentile) dose is shown along with the static value. (b) The minimum (1st percentile) dose is shown along with the static value. (c) The median dose is shown along with the static value. (d) The mean dose is shown along with the static value.

The probability density distributions of maximum, minimum, median, and mean dose values within the 90% static isodose curve for 1000 trials of single fraction treatments are shown in Fig. 9. The maximum dose (99th percentile) is defined as maximum dose that covers 1% of the region of interest. In the moving trials, the maximum dose peaks slightly higher than the static case, with a longer probability tail in the overdose region. The minimum dose (1st percentile) is defined as the minimum dose that covers 99% of the region of interest. The distribution shows a high probability of underdosage, compared to the static value, in the region of interest, with the peak in the probability density distribution around 84%. The median and mean dose probability distributions both show a lower peak than the static case. The peaks in these distributions are around 93%, compared to approximately 95% for no motion. From these probability density distributions we can conclude that, in the presence of motion, the dose distribution in the region of interest, the high‐dose region, will likely be more spread out than the static case. The maximum dose has the highest probability to be close to the static case with a small (~5%) chance of being at least 4% higher. The minimum dose has a high probability (~95%) of an underdose, with the most likely scenario being an underdose by almost 6% of the static case value. In addition, the mean and median doses will very likely be around 2% lower compared to the static case, with a low probability (~5%) of being higher than the static case. This compares well with the chamber measurements that showed a mean dose within 1% to 2% of the static value.^(7)^

**Figure 10:**
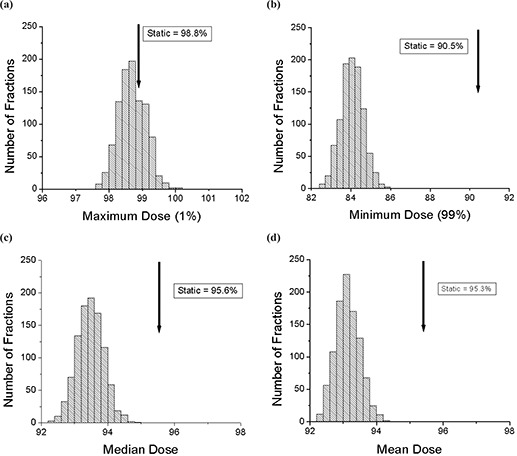
The histograms for 1000 trials of 30‐fraction treatments with motion. All the values are calculated within the region of interest (static 90% isodose curve). (a) The maximum (99th percentile) dose is shown along with the static value. (b) The minimum (1st percentile) dose is shown along with the static value. (c) The median dose is shown along with the static value. (d) The mean dose is shown along with the static value.

The probability density distributions of maximum, minimum, median, and mean dose values within the 90% static isodose curve for 1000 trials of 30‐fraction treatments are shown in Fig. 10. The definitions are the same as above. These distributions are all sharply peaked, as is expected from our finding of a small (<1%) standard deviation for every pixel. The results are similar to the single‐fraction case. The peak of the maximum dose is just below (<0.5%) that of the static case. The minimum dose distribution peaks well below (~6%) that of the static case. Both the median and mean distributions peak below (~2%) those of the static value. The dose distributions from several 30‐fraction deliveries will not vary much due to the motion. However, there is slight underdosing of the region of interest, by several percent, in the presence of motion, even for the 30‐fraction delivery.

The visual method of choosing the eight sample points in the breathing cycle did not introduce any significant uncertainty in this study. The mechanical wheel that drives the sinusoidal motion of the platform was broken into eight sections. If one looks at the wheel as a 360° circle, each section is 45°. The period of the motion was approximately 4 s, which implies that a new section passed every 0.5 s. To estimate the uncertainty in the initial phase, we determined that we were able to engage the beam at least within ±0.25 s, or 22.5°, along the mechanical wheel. Previous simulations suggest that a smooth function describes the change in dose with change in initial phase.^(11)^ We concluded that since the change in dose is smooth and that “beam on” occurred within 0.25 s of the appropriate time, the uncertainty in the initial phase angle was irrelevant for this study.^(7)^

The results from the film measurements included several sources of uncertainty. The film processing and the conversion from optical density to dose introduced uncertainties that are inherent in EDR2 film. In a recent study, the variation in the sensometric curve for EDR2 film was found to be less than 4% for a 6‐MV photon beam.^(20)^ The alignment error, described above, was negligible due to the field edge alignment postdigitization.

## IV. CONCLUSION

The interplay effect between organ motion and IMRT delivery with a dynamic MLC was studied using Kodak EDR2 film and a platform moving in a sinusoidal pattern. It was determined that possible hot/cold spots in the target region due to this effect become mostly blurred out over 30 fractions. However, there is the possibility of underdosing the tumor by several percent in the presence of motion. We would also like to point out that patient breathing is, in general, not as regular in amplitude and frequency as the moving platform in our experiment. In order to ensure regularity, a method of breath coaching may have to be used with patients.^(^ ^21^ ^,^ ^22^ ^)^ These conclusions are in agreement with our previous study using an ion chamber for measurement.

## ACKNOWLEDGMENT

This work was partially supported by grants from Varian Medical Systems, Inc., and the Whitaker Foundation.
